# Supplementary material for: Thermal fluctuations of the lipid membrane determine particle uptake into Giant Unilamellar Vesicles
Source: Nat Commun. 2023 Jan 4;14:65. doi: 10.1038/s41467-022-35302-5 (PMC9813155; doi:10.1038/s41467-022-35302-5)
Supplement: Supplementary file 1 — Supplementary Information [file 41467_2022_35302_MOESM1_ESM.pdf]

Supplementary Information for

Thermal fluctuations of the lipid membrane determine particle uptake into Giant Unilamellar Vesicles

Yareni A. Ayala[1], Ramin Omidvar[2,3], Winfried Römer[2,3,4], Alexander Rohrbach *[,1,3]

[1] Laboratory for Bio- and Nano-Photonics, Department of Microsystems Engineering - IMTEK, University of Freiburg, Georges-Köhler-Allee 102, 79110, Freiburg, Germany

[2] Faculty of Biology, University of Freiburg, Schänzlestraße 1, 79104, Freiburg, Germany

[3] Signalling Research Centres BIOSS and CIBSS, University of Freiburg, Schänzlestraße 18, 79104, Freiburg, Germany

[4] Freiburg Institute for Advanced Studies (FRIAS), University of Freiburg, Albertstraße 19, 79104, Freiburg, Germany

* Correspondence: rohrbach@imtek.de

Alexander Rohrbach

* Correspondence: rohrbach@imtek.de

This PDF file includes:

Supplementary notes
Supplementary Figures S1 to S19
Supplementary References

Supplementary note 1: Membrane fluctuations with and without bead

The Supplementary Figs. S1 and S2 represent an image composition from two movies of GUV fluctuations without bead indentation (Supplementary Figs. S1 a and S2 a) and with bead indentation (Supplementary Figs. S1 b and S2 b). Three horizontal and one vertical line scans represent kymographs of the fluctuating, fluorescent membrane. The total widths $\sqrt{\sum\langle|h(q)|^2\rangle}$ of all fluctuation modes *q* as well as the mean displacements from the bead indentation can be roughly estimated from the fluorescence movies. However, the measurement of precise fluctuations in time and space is only possible through interferometric thermal noise tracking.

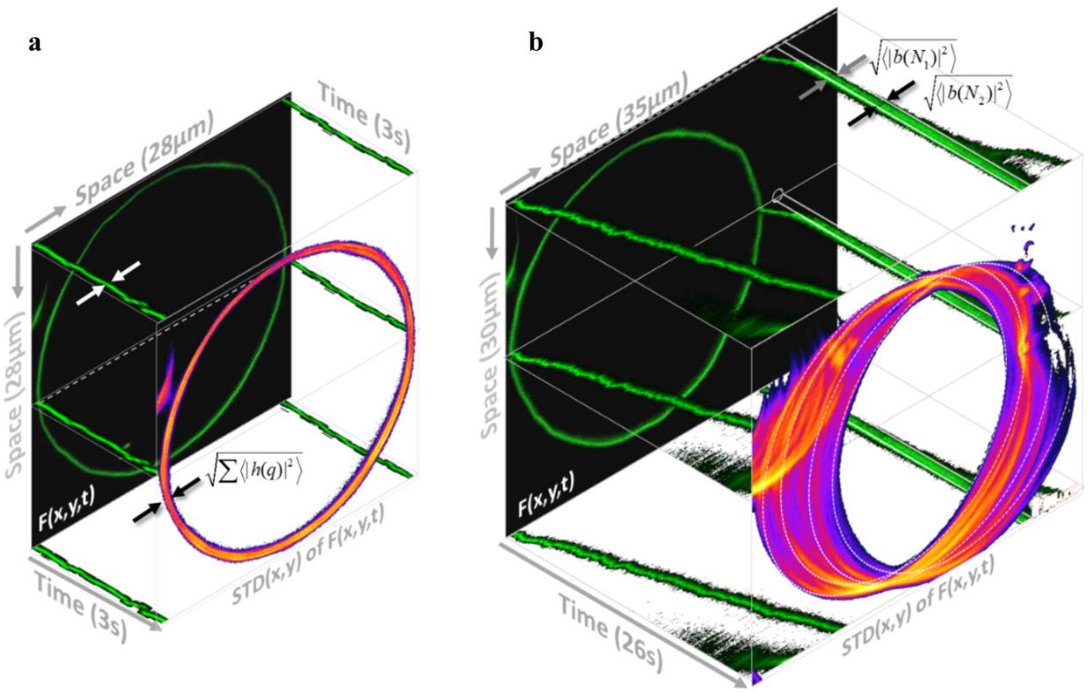

Supplementary Fig. S1: Image composition corresponding to Supplementary Video 1 of flaccid GUV showing membrane fluctuations and deformations. Fluorescence distribution $F(x,y,t_0)$ of membrane (in green) changing over time t_0, kymograph $F(x,y_0,t)$ along horizontal direction (dashed line) and time projection of fluorescence standard deviation $STD(x,y)$ shown in fire colors. **a** Membrane fluctuations of the GUV without the bead. **b** Membrane fluctuations of the GUV approaching the static, trapped bead.

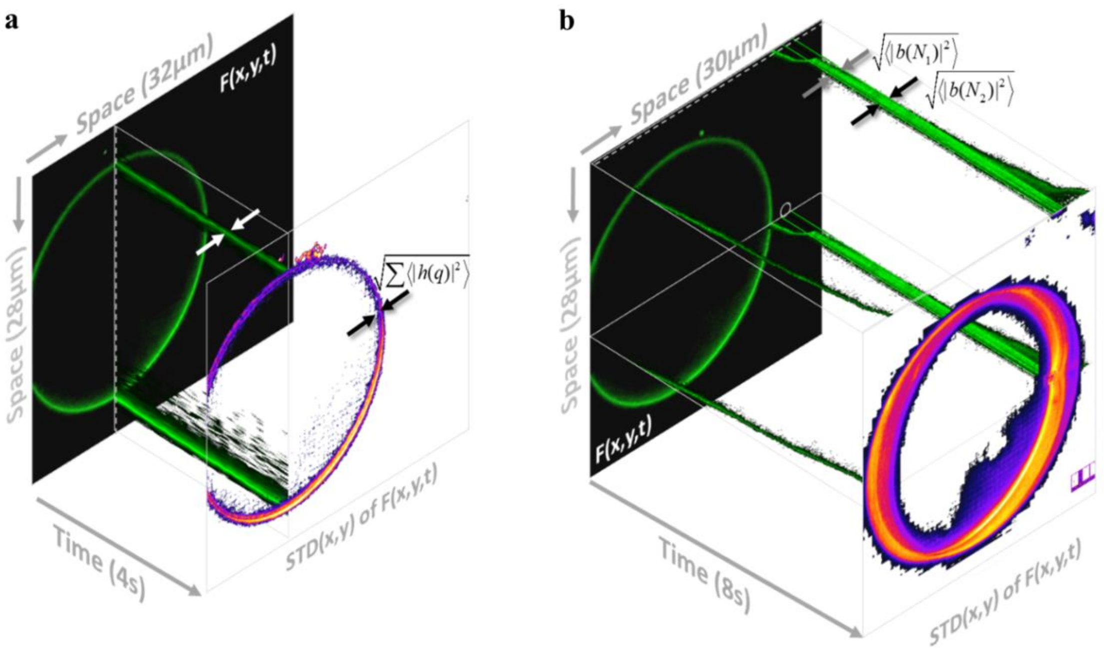

Supplementary Fig. S2: Image composition corresponding to Supplementary Video 2 of tense GUV showing membrane fluctuations and deformations. Fluorescence distribution $F(x,y,t_0)$ of membrane (in green) changing over time t_0, kymograph $F(x,y_0,t)$ along horizontal direction (dashed line) and time projection of fluorescence standard deviation $STD(x,y)$ shown in fire colors. **a** Membrane fluctuations of the GUV without the bead. **b** Membrane fluctuations of the GUV approaching the static, trapped bead.

Fluctuations from planar and spherical membranes

Fluctuation variance of N modes, where each mode couples independently of the trap

Membrane stiffness $\kappa_m(\mathrm{n},\mathrm{K},\sigma) = \frac{1}{q_0}\left(K\cdot q(\mathrm{n})^4 + \sigma\cdot q(\mathrm{n})^2\right)$ $\quad \left\langle |\, b(N,\mathrm{K},\sigma,\kappa_{opt})\,|^2 \right\rangle = \frac{k_B T}{\kappa_{opt} + \left(\sum_{n=n_{mim}}^{N} \frac{1}{\kappa_m(\mathrm{n},\mathrm{K},\sigma)}\right)^{-1}}$

Membrane by spherical harmonics with m=0 $\quad Y(\mathrm{n},\theta) = \sqrt{\frac{2n+1}{4\pi}}\cdot Leg(\mathrm{n},\cos(\theta))$ $\quad \theta = -\pi, -\pi+0.01\ldots\pi$

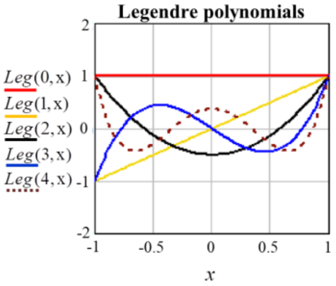

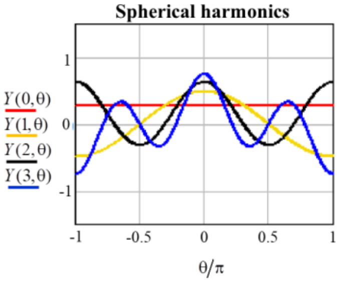

Single mode mean fluctuations width for $k_BT/2$: $\left\langle \left| b(\mathrm{n},K,\sigma,\mathrm{R}_g)\right|^2 \right\rangle = R_g^2 \frac{k_B T}{K\cdot(\mathrm{n}+2)\cdot(\mathrm{n}-1)\cdot\mathrm{n}\cdot(\mathrm{n}+1) + \sigma\cdot R_g{}^2\cdot(\mathrm{n}+2)\cdot(\mathrm{n}-1)}$

All mode mean fluctuations width for $k_BT/2$: $\left\langle \left| b(N,\mathrm{K},\sigma,\mathrm{R},\kappa_{opt})\right|^2 \right\rangle = \frac{k_B T}{\kappa_{opt} + \left(\frac{1}{k_B T}\sum_{n=n_{mim}}^{N}\left\langle \left| b(\mathrm{n},K,\sigma,\mathrm{R}_g)\right|^2\right\rangle\right)^{-1}}$

Experimental input values

	Tense	Flaccid
GUV radii:	$R_{g1} = 7.5\mu m$	$R_{g2} = 13\mu m$
Bending rigidity:	$K_1 = 12k_B\mathrm{T}$	$K_2 = 1.6k_B\mathrm{T}$
Tension:	$\sigma_1 = 300\,k_B\mathrm{T}/\mu m^2$	$\sigma_2 = 15\,k_B\mathrm{T}/\mu m^2$
Trap stiffness:	$\kappa_{opt,1} = 60\,pN/\mu m$	$\kappa_{opt,2} = 15\,pN/\mu m$

Comparison with flat membrane

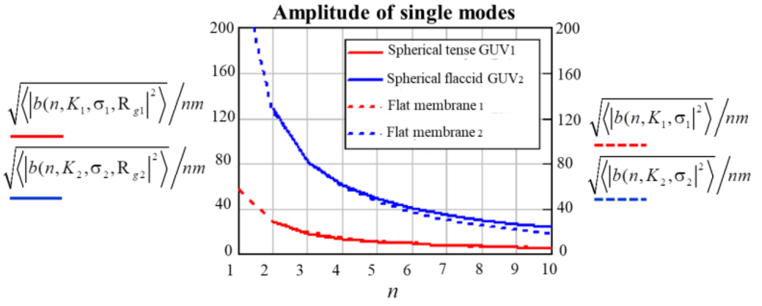

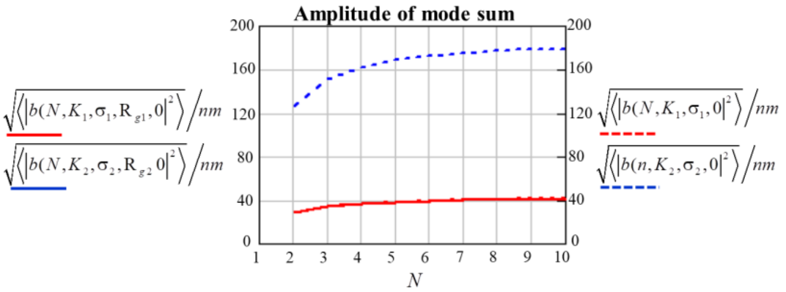

Supplementary Fig. S3: Comparison of fluctuation amplitudes for spherical membranes (GUVs) and flat membranes. Development of a spherical membrane into spherical harmonic functions lead to very similar fluctuation amplitudes as for flat membranes [1]. Top graph: ½ k_BT amplitude for single modes. Bottom graph: $n/2$ k_BT amplitude for mode sum. The GUV radii, bending stiffnesses K and surface tensions σ are chosen to correspond to the experimental data.

Supplementary note 2: fluctuation width of planar and spherical membranes

In Supplementary Fig. S3 the square roots of the fluctuation variances $\left\langle |b(n,K,\sigma)|^2 \right\rangle$ and $\left\langle |b(n,K,\sigma,R_g)|^2 \right\rangle$ of single fluctuation modes are shown (upper graph), as well as those of the fluctuation variances $\left\langle |b(N,K,\sigma|^2 \right\rangle$ and $\left\langle |b(N,K,\sigma,R_g)|^2 \right\rangle$ of the sum of *N* independent coupling modes (bottom graph). Here the dependence on R_g represents the radial spherical fluctuations. For the experimental relevant parameters, the difference in the fluctuation widths is negligible.

Supplementary note 3: change of relaxation times by adding or suppressing fluctuation modes

The relaxation time of summing up the modes depends on the number ranging from n_0 to N_{mx}. The change in the slope depends on the relation between the optical trap stiffness κ_{opt} and the membrane parameters like GUV radius R_g , bending rigidity *K* or membrane tension *σ*. This is illustrated for five examples in the following Supplementary Fig. S4:

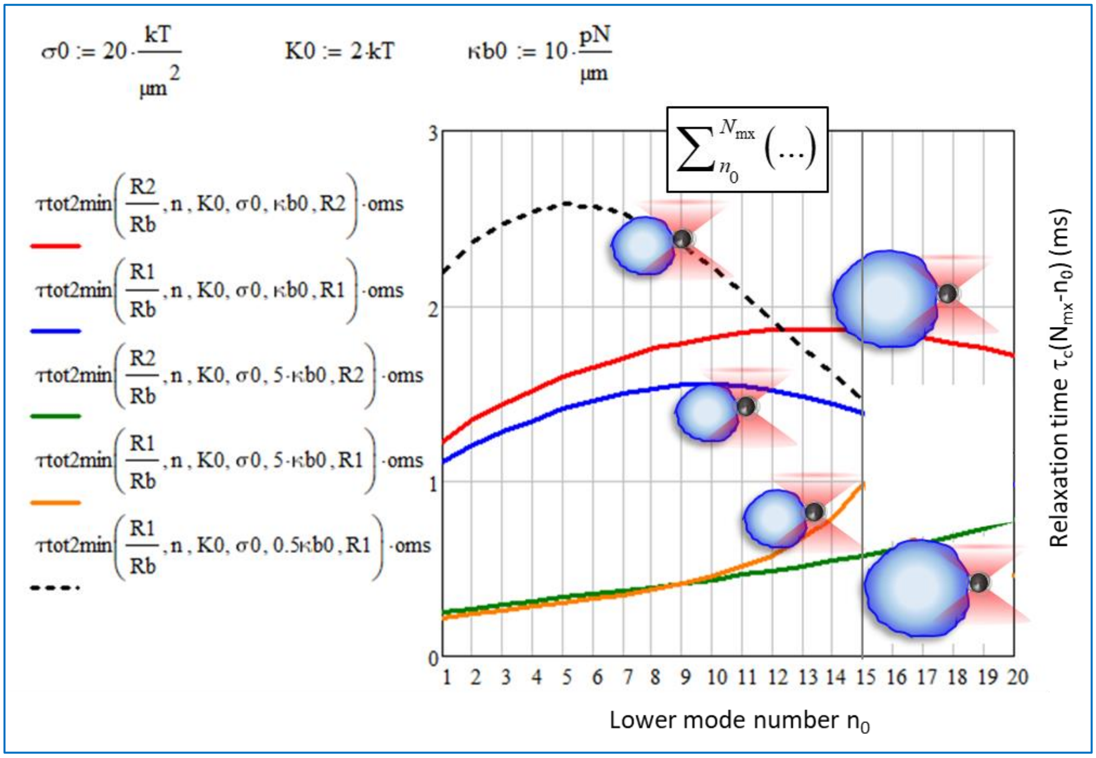

Supplementary Fig. S4: The relaxation times (*AC* times τ_c) of a bead in contact with the membrane increase with increasing lower mode number n_0, corresponding to subsequent exclusion of lower modes. This is true for most, but not all GUV / membrane parameters and trap stiffnesses (see the case for a weak optical trap, black dotted line). The maximum mode numbers are $N_{mx1} = R_{g1}/R_b = 15$ and $N_{mx2} = R_{g2}/R_b = 26$.

Supplementary note 4: Frequency dependent, complex value response functions

For the parallel connection between bead and membrane the Langevin equation in Fourier space is $\tilde{b}(\omega)(\kappa_{opt}+i\omega\gamma_{bd})+\tilde{b}(\omega)(\kappa_{mem}+i\omega\gamma_{mem})=\tilde{F}_{th}(\omega)$. Solving for the displacement $\tilde{b}=\alpha_{tot}\cdot\tilde{F}_{th}$ results in

$$\tilde{F}_{th}(\omega)/\tilde{b}(\omega)=\frac{1}{\alpha_{tot}(\omega,N)}=(\kappa_{opt}+i\omega\gamma_{bd})+(\kappa_{mem}+i\omega\gamma_{mem})=\frac{1}{\alpha_{bd}(\omega)}+\frac{1}{\alpha_{mem}(\omega,N)}. \quad (1)$$

Hence, the response function of both the viscoelastic behavior of the optically trapped bead and all membrane modes (serially connected) can be written as:

$$\alpha_{tot}(\omega,N)=\frac{1}{\kappa_{tot}+i\omega\gamma_{tot}}=\frac{1}{(\kappa_{opt}+i\omega\gamma_{bd})+(\kappa_{mem}+i\omega\gamma_{mem})}=\frac{1}{(\kappa_{opt}+i\omega\gamma_{bd})+\frac{1}{\sum_N\left(\kappa_{qm}+i\omega\gamma_{qm}\right)^{-1}}}$$
$$=\frac{1}{1/\alpha_{bd}+1/\alpha_{mem}}=\frac{1}{1/\alpha_{bd}+1/\sum\alpha_{qm}}=\left(\left(\kappa_{opt}+i\omega\gamma_{bd}\right)+\frac{1}{\alpha_{Rqm}(\omega)-i\omega\cdot\alpha_{Iqm}(\omega)}\right)^{-1} \quad (2)$$

with membrane response function $\alpha_{mem}(\omega,N)=\sum_N\left(\kappa_{qm}+i\omega\gamma_{qm}\right)^{-1}=\sum_N\frac{\left(\kappa_{qm}-i\omega\gamma_{qm}\right)}{\left(\kappa_{qm}{}^2+\omega^2\gamma_{qm}{}^2\right)}$

$$=\sum_N\frac{\kappa_{qm}}{\left(\kappa_{qm}{}^2+\omega^2\gamma_{qm}{}^2\right)}-i\omega\cdot\sum_N\frac{\gamma_{qm}}{\left(\kappa_{qm}{}^2+\omega^2\gamma_{qm}{}^2\right)}=\alpha_{Rqm}(\omega)-i\omega\cdot\alpha_{Iqm}(\omega).$$

The squared modulus of the total response function is (see Supplementary Fig. S5 b):

$$\left|\alpha_{tot}(\omega,N)\right|^2=\frac{1}{(\kappa_{opt}+\kappa_{mem})^2+\omega^2(\gamma_{bd}+\gamma_{mem})^2}=\frac{1/(\gamma_{bd}+\gamma_{mem})^2}{\left(\frac{\kappa_{bd}+\kappa_{mem}}{\gamma_{bd}+\gamma_{mem}}\right)^2+\omega^2}. \quad (3)$$

The total response function can be split into a real part and imaginary part (see Supplementary Fig. S5):

$$\alpha_{tot}(\omega,N)=\alpha_{R,tot}(\omega)+i\omega\cdot\alpha_{I,tot}(\omega)=\frac{1}{\kappa_{tot}+i\omega\gamma_{tot}}=\frac{\kappa_{tot}}{\kappa_{tot}{}^2+\omega^2\gamma_{tot}{}^2}-i\omega\frac{\gamma_{tot}}{\kappa_{tot}{}^2+\omega^2\gamma_{tot}{}^2}$$
$$=\frac{1}{(\kappa_{bd}+\kappa_{mem})+i\omega(\gamma_{bd}+\gamma_{mem})}=\frac{\kappa_{bd}+\kappa_{mem}}{(\kappa_{bd}+\kappa_{mem})^2+\omega^2(\gamma_{bd}+\gamma_{mem})^2}-i\omega\frac{(\gamma_{bd}+\gamma_{mem})}{(\kappa_{bd}+\kappa_{mem})^2+\omega^2(\gamma_{bd}+\gamma_{mem})^2}. \quad (4)$$

In the high-frequency case $\omega>\omega_{opt}=\frac{\kappa_{opt}}{\gamma_{bd}}$, i.e. on short timescales, we find:

$$\alpha_{tot}(\omega>\omega_{opt},N)=-i\omega\frac{\gamma_{tot}}{\kappa_{tot}{}^2+\omega^2\gamma_{tot}{}^2}=-i\omega\frac{(\gamma_{bd}+\gamma_{mem})}{(\kappa_{opt}+\kappa_{mem})^2+\omega^2(\gamma_{bd}+\gamma_{mem})^2}\approx-i\frac{1}{\omega(\gamma_{bd}+\gamma_{mem})}. \quad (5)$$

If the membrane viscous drag is much larger than that of the bead $\gamma_{mem}\gg\gamma_{bd}$ (Supplementary Fig. S5 c) the response function reads:

$$\alpha_{tot}(\omega>\omega_{opt},N)\approx-i\frac{1}{\omega(\gamma_{bd}+\gamma_{mem})}\approx-\frac{i}{\omega\gamma_{mem}}=-\frac{i}{\omega}\left(\sum_q\frac{1}{\gamma_{qm}}\right)^{-1}. \quad (6)$$

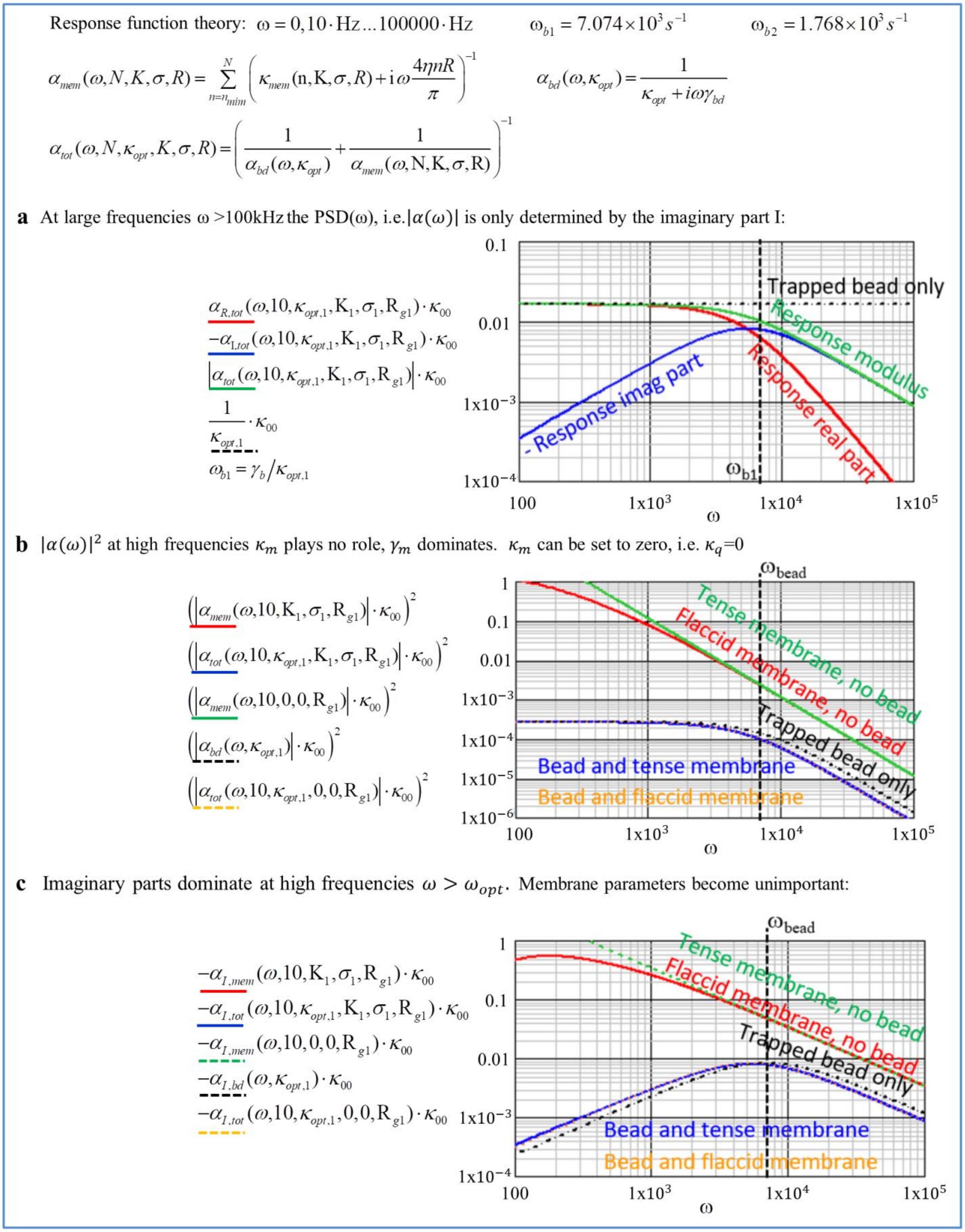

Supplementary Fig. S5: Comparison of response functions for a trapped bead, for the membrane and the combination of bead and membrane. a Decaying real part (red), increasing imaginary part (blue) and slowly decreasing modules (green) of the total response function from the bead connected to membrane. The case of a trapped bead only is shown in black. **b** The power spectral density, being proportional to $|\alpha(\omega)|^2$, reveals the same high frequency dependence for a tense and an extremely flaccid membrane ($K\to0$, $\sigma\to0$), which is mainly determined by the viscous drag. **c** The imaginary

parts dominate the fluctuation response in the high-frequency case, where membrane parameters (K,σ) become unimportant.

Hence, the total response is purely determined by friction $\left|\alpha_{tot}(\omega,N)\right|^2 = \frac{1}{\kappa_{tot}{}^2+\omega^2\gamma_{tot}{}^2} \overset{\omega>\omega_{opt}}{=} \frac{1}{\omega^2\gamma_{tot}{}^2} = \frac{1}{\omega^2(\gamma_{bd}+\gamma_{mem})^2}$

Without bead and optical trapping forces the bead displacement is:

$$\tilde{b}(\omega,\kappa_{opt}=0) = \frac{1}{\kappa_{mem}+i\omega\gamma_{mem}} \cdot \tilde{F}_{th}(\omega) = \sum_q \tilde{b}_q(\omega,0) = \sum_q \frac{1}{\kappa_{qm}+i\omega\gamma_{qm}} \cdot \tilde{F}_{th}(\omega). \quad (7)$$

If κ_{mem} = 0 or in the high-frequency case, the membrane response $\kappa_{mem}+i\omega\gamma_{mem} = \left(\sum_q \frac{1}{\kappa_{qm}+i\omega\gamma_{qm}}\right)^{-1}$ becomes $\gamma_{mem} = \left(\sum_q \frac{1}{\gamma_{qm}}\right)^{-1}$.

Supplementary note 5: Model for membrane deformation by a particle

Bending and stretching the membrane costs energy (and force), which are proportional to K for bending and to σ for stretching lipids relative to each other, as described by the Helfrich free energy [2,3]. Here $h(\theta)$ is the height function describing the shape of the membrane, which can be described as a function of the polar angle relative to the axis of indentation:

$$G_{mem}\left(h(\theta)\right) = \frac{1}{2}\int_0^{2\pi}\int_0^{\theta}\left[K\cdot(\nabla^2 h(r,\Theta,\varphi))^2 + \sigma\cdot(\nabla h(r,\Theta,\varphi))^2\right]d\Theta d\varphi\,. \quad (8)$$

Using fluorescence microscopy, one can see two different types of GUV deformations [4]. As sketched in Fig. 2e and Supplementary Fig. S6, there is a global deformation from the round GUV with radius R_g to an ellipsoidally deformed GUV (with half axes a and c) caused by force F_{opt} of the trapped particle. This deformation, defined by the mean free energy $G^{glo}(h_{gl})$, can be quantified by the global indentation distance h_{gl}. In addition, there is a local deformation caused by the bead, which generates a toroidal membrane indentation with torus minor radius R_c. Two circles with appropriate radii R_c fit well to fluorescence indentation profiles of the GUV (Fig. 2e bottom).

The local indentation distance is denoted as h_{lo} and is defined by the local free energy $G^{loc}(h_{lo})$ of the membrane. The corresponding damped (non-linear) springs form a serial connection with each other, but a parallel connection with the optical trap, as outlined in Fig. 2e bottom and Supplementary Fig. S6. Therefore, the total free energy $G_{mem}(d)$ is explored by the addition of the local and global indentation lengths $h_{lo}+h_{gl} = d(h_{lo})$ according to

$$G_{mem}\left(h_{lo}+h_{gl}\right) = \begin{cases} G^{loc}(h_{lo}) + G^{glo}(h_{gl}) & \text{if } d < d_{up} \\ G_{tube}\left(d(h_{lo}+h_{gl})\right) & \text{if } d > d_{up} \end{cases} \quad (9)$$

The indentation function $d(h_{lo}) = h_{lo}+h_{gl} = h_{lo}+z(h_{lo})h_{lo}$ can be found by the minimum of the potentials $\left(G^{loc}(h_{lo})+G^{glo}(h_{gl})\right)\rightarrow \min$ or for the zeros $z(h_{lo})$ of the corresponding force difference $F^{loc}(h_{lo}) - F^{glo}(h_{gl}) = 0$, such that $F^{loc}(h_{lo}) = F^{glo}(z(h_{lo}))$, as required for a serial connection of forces.

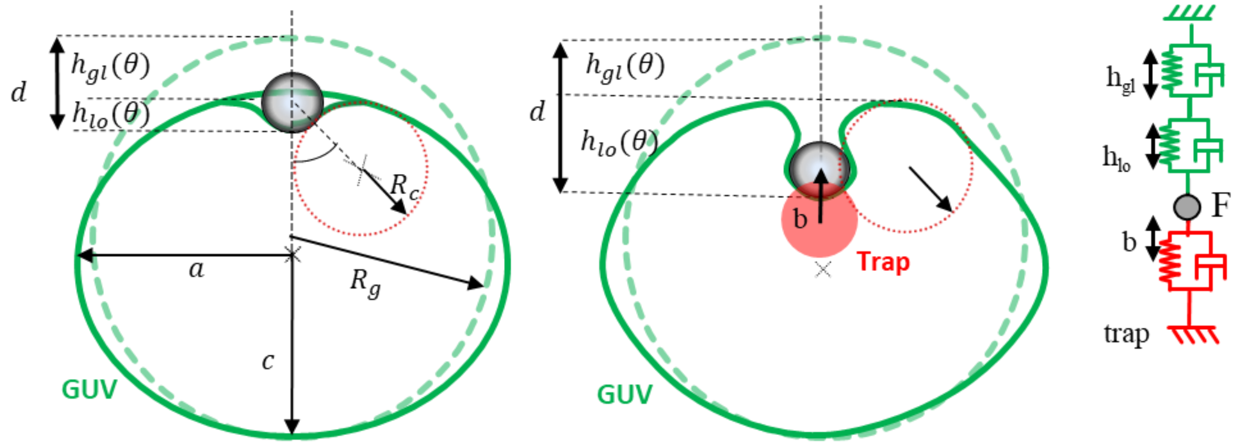

Supplementary Fig. S6: Deformation schematic for the bead indentation into a GUV. A global deformation results from the change of a spherical vesicle (see green dashed line) with radius R_g to an elliptically deformed vesicle with half axis a and c. For this situation, the global indentation height of the bead is $h_{gl}(\theta)$, which is a function of the polar angle θ. The additional local deformation results in a local shape, which can be described by the surface of a torus with a minor torus radius R_c (indicated by the red dotted lines). It is assumed that the torus radius R_c is not changing during the indentation expressed by the local height $h_{lo}(\theta)$.

The Helfrich free energy [4] in Eq. (9) can be split into global and local terms for stretching and bending (see Supplementary Eqs. (11) - (15)), such that

$$G_{mem}\left(d(h_{lo})\right) = \left(-G_{ad}(h_{lo}) + G_{ben}^{loc}(h_{lo}) + G_{str}^{loc}(h_{lo})\right) + \left(G_{ben}^{glo}(h_{gl}) + G_{str}^{glo}(h_{gl})\right), \tag{10}$$

here, $G_{ad}(h_{lo}) = 2\pi \cdot w \cdot R_b \cdot h_{lo}$ is the adhesion energy with surface energy density w, which will become important for specific biological binding partners used in the study.

In the following we explain how to calculate the local bending and stretching energies and the global bending and stretching energies depending on their indentation lengths h_{gl} and h_{lo}, respectively.

The global deformation

It considers the change of the spherical GUV of radius R_g to an oblate ellipsoid shaped GUV with half axis a, and c. From these two half-axis, the ellipticity eps(h) is defined and thereby the increase of the GUV surface area, resulting in **the global stretching energy** with surface tension σ

$$G_{str}^{glo}(h_{gl}, R_g) = \sigma \cdot \left(A_{ell}(\tfrac{1}{2}h_{gl}, R_g) - A_{sph}(R_g)\right). \tag{11}$$

The global bending energy $G_{ben}^{glo}(h_{gl})$ is proportional to the bending rigidity *K,* a material constant of the membrane, which can be tuned by different lipid and protein compositions. It is defined by the surface area of the GUV and the curvatures κ_1 and κ_2 in orthogonal directions:

$$G_{ben}^{glo}(h_{gl},R_g) = \tfrac{1}{2}K\cdot\left(A_{ell}(h_{gl},R_g)\cdot\left(\kappa_1(h_{gl})+\kappa_2(h_{gl})\right)^2 - A_{sph}(R_g)\cdot\frac{4}{R_g^{\ 2}}\right). \quad (12)$$

Experimental membrane parameters (σ_1, K_1, R_{g1}), ($0.5\sigma_1$, K_1, R_{g1}), (σ_2, K_2, R_{g2}) are chosen as shown in Supplementary Fig. S7.

Experimental values for tense and flaccid vesicles

Trap stiffness: $\kappa_{opt,1} = 60\,pN/\mu m$, $\kappa_{opt,2} = 15\,pN/\mu m$ GUV radii: $R_{g1} = 7.5\mu m$, $R_{g2} = 13\mu m$

Relaxation time in trap $\tau_{01} = \frac{\gamma_{bd}}{\kappa_{opt,1}} = 0.141ms$, $\tau_{02} = \frac{\gamma_{bd}}{\kappa_{opt,2}} = 0.565ms$

Membrane parameters from measured tether radii $R_{t1} = 0.14\mu m$, $R_{t2} = 0.23\mu m$ and forces $F_{t1} = 2.1pN$, $F_{t2} = 0.17\,pN$:

$\sigma_1 = 300\,k_B T/\mu m^2$ $K_1 = 12\,k_B T$ $\Lambda(K_1,\sigma_1) = 0.2\,\mu m$ $R_t(K_1,\sigma_1) = 141\,nm$ $F_t(K_1,\sigma_1) = 2.1\,pN$

$\sigma(F_{t1},R_{t1}) = 298.416\ k_B T/\mu m^2$ $K(F_{t1},R_{t1}) = 11.698\,k_B T$ $\Lambda(F_{t1},R_{t1}) = 0.198\,\mu m$

$\sigma_2 = 15\,k_B T/\mu m^2$ $K_2 = 1.6 k_B T$ $\Lambda(K_2,\sigma_2) = 0.327\mu m$ $R_t(K_2,\sigma_2) = 231\,nm$ $F_t(K_2,\sigma_2) = 0.174\,pN$

$\sigma(F_{t2},R_{t2}) = 14.705\ k_B T/\mu m^2$ $K(F_{t2},R_{t2}) = 1.556\,k_B T$ $\Lambda(F_{t2},R_{t2}) = 0.325\,\mu m$

Surface energy density: $w_1 = 20\,k_B T/\mu m^2$

Supplementary Fig. S7: Experimental values used for the simulations. For exemplary calculations in MathCad 15 we used two different trap stiffnesses $\kappa_{opt,1}$ and $\kappa_{opt,2}$, two different membrane surface tensions σ_1 and σ_2, and two different bending rigidities K_1 and K_2.

In Supplementary Fig. S8 the global stretching and bending energies are compared in the upper graph and the corresponding forces in the bottom graph. It can be seen that the global stretching energy rises stronger than the global bending energy at low indentations h_{gl}, whereas bending energies and forces dominate for larger h_{gl} values.

Surfaces are in μm^2, when one half axis is shortened by h_{lo} :

$$A_{sph}(R_g) = 4\pi R_g^{\ 2}$$

$$A_{ell}(h_{lo}, R_g) = 2\pi a^2(h_{lo}, R_g)\left[1 + \left(\frac{c(h_{lo}, R_g)}{a(h_{lo}, R_g)}\right)^2 \cdot \mathrm{arctan}(h_{lo}, R_g)\right] \quad \text{with } \mathrm{arctan}(h_{lo}, R_g) = \begin{cases} \dfrac{\mathrm{arctan}(\mathrm{eps}(h_{lo}, R_g))}{\mathrm{eps}(h_{lo}, R_g)} & \text{if } h_{lo} \neq 0 \\ 1 & \text{otherwise} \end{cases}$$

$$A_{ell}(h_{lo}, R_g) = \left(2\pi a^2(h_{lo}, R_g) + 2\pi c^2(h_{lo}, R_g) \cdot \mathrm{arctan}(h_{lo}, R_g)\right)$$

Global stretching energy as a function of indentation height h_{gl}, which is $h_{lo} = \frac{1}{2} h_{gl}$

$$G_{str}^{glo}(h_{gl}, \sigma, R_g) = \sigma \cdot \left(A_{ell}(\tfrac{1}{2} h_{gl}, R_g) - A_{sph}(R_g)\right)$$

Global numerical stretching force: $F_{str}^{glo}(h_{gl}, \sigma, R_g) = \dfrac{d}{dh_{gl}} G_{str}^{glo}(h_{gl}, \sigma, R_g)$

Membrane deformation model without mode fluctuations

1. Global deformations $\quad R_{g2} = 13\mu m \quad R_g = 10\mu m \quad h_{lo} = 0, 0.01\mu m ... R_{g2}/2 \quad h_{gl} = 0, 0.001\mu m ... R_{g2}/2$

Surface of oblate ellipsoid with $a = b > c$ and $a = R_g + h_{lo}$ and $c = R_g - h_{lo}$

Radii and half axes if GUV volume is assumed to be constant: $R_g^{\ 3} = a^2 c$ with $a = R_g\sqrt{R_g/c}$

$$a(h_{lo}, R_g) = R_g\sqrt{\frac{R_g}{(R_g - h_{lo})}} \qquad c(h_{lo}, R_g) = R_g - h_{lo} \qquad \text{ellipticity } eps(h_{lo}, R_g) = \sqrt{1 - \left(\frac{c(h_{lo}, R_g)}{a(h_{lo}, R_g)}\right)^2}$$

Bending energy: $G_{ben}(\kappa_1, \kappa_2, A, K) = K/2(\kappa_1 + \kappa_2)^2 A$

$$G_{ben}^{sph}(K, R_g) = G_{ben}\left(\frac{1}{R_g}, \frac{1}{R_g}, A_{sph}(R_g), K\right) \qquad G_{ben}^{ell}(h_{lo}, K, R_g) = G_{ben}\left(\frac{c(h_{lo}, R_g)}{a^2(h_{lo}, R_g)}, \frac{a(h_{lo}, R_g)}{c^2(h_{lo}, R_g)}, A_{ell}(h_{lo}, R_g), K\right)$$

Global bending energy: $G_{ben}^{glo}(h_{gl}, K, R_g) = G_{ben}^{ell}(h_{gl}, K, R_g) - G_{ben}^{sph}(K, R_g)$

Global numerical bending force: $F_{ben}^{glo}(h_{gl}, K, R_g) = \dfrac{d}{dh_{gl}} G_{ben}^{glo}(h_{gl}, K, R_g)$

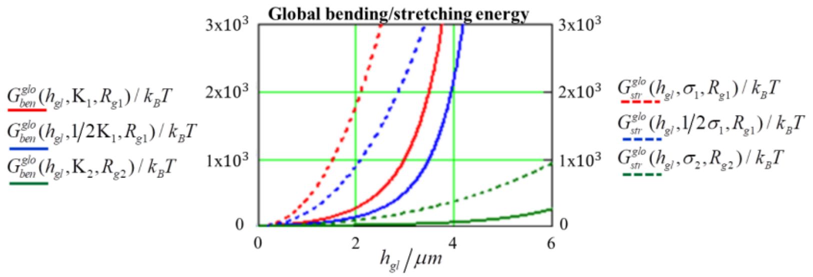

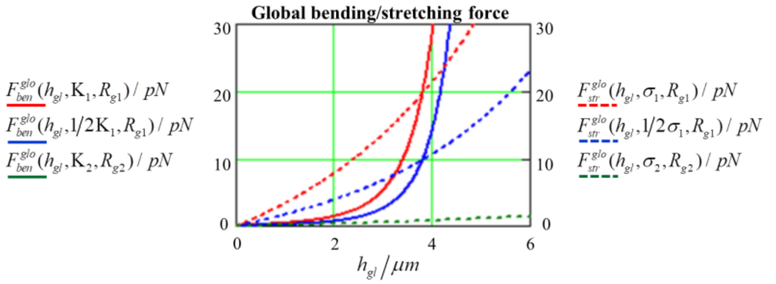

Supplementary Fig. S8: Global stretching energy/force and global bending energy/force. Global energies (in $k_B T$) and forces (in pN) for stretching and bending are compared for three GUVS, with different radii (R_{g1} and R_{g2}), bending rigidities (K_1 and K_2) and surface tensions (σ_1 and σ_2) as a function of global indentation height h_{gl} in µm. The sum of both global deformation energies is shown up to 3000 $k_B T$.

Supplementary Fig. S9 shows the sums of global energies and global forces. Hence, the global deformation energy rises smoothly due to initial stretching and then more steeply due to the high bending energy costs.

Total global energy: $G^{glo}(h_{gl},\sigma,K,R_g) = G^{glo}_{ben}(h_{gl},K,R_g)+G^{glo}_{str}(h_{gl},\sigma,R_g)$

Total global force: $F^{glo}(h_{gl},\sigma,K,R_g) = F^{glo}_{ben}(h_{gl},K,R_g)+F^{glo}_{str}(h_{gl},\sigma,R_g)$

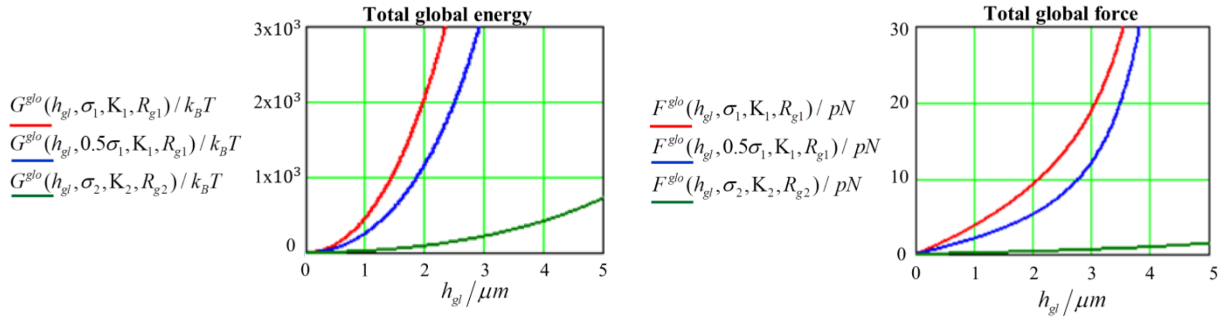

Supplementary Fig. S9: Sum of energies and forces for global stretching and global bending.

The local deformation

It considers the energy costs for bending and stretching the membrane when a flat circular area is deformed into a spherical surface area defined by the radius of the bead and a torus surface area defined by concave (inner) torus radius, as shown in Supplementary Fig. S10. By analyzing the fluorescence indentation movies (see Fig. 3a), we found that the torus radius is approximately 2.5 times the bead radius for the smaller, tense GUV ($2R_{g1}$ = 15µm) and is approximately independent of the indentation height, i.e. $R_{C1} \approx 2.5 \cdot R_b$. For the larger, flaccid GUV ($2R_{g2}$ = 26µm), the torus radius is approximately five times the bead radius i.e. $R_{C2} \approx 5 \cdot R_b$, once more approximately independent of the indentation height. As sketched in Supplementary Fig. S10, the local indentation height is a function of angle θ according to $h_{lo}(\theta) = p \cdot (R_b + R_c) = (1-\cos(\theta))(R_b + R_c)$ and where $q = \sin(\theta)$.

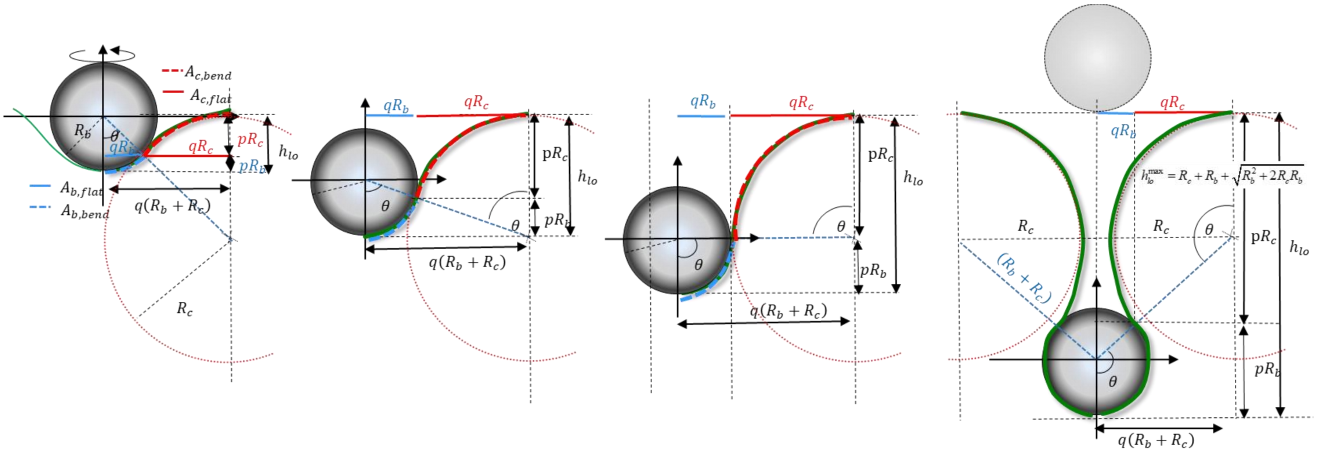

Supplementary Fig. S10: Change in GUV surface area as a function of local indentation height. Schematics of three different stages of bead indentation up to a maximal penetration depth, which is assumed to be reached when area increase goes back to zero $\Delta A(h_{lo}) \approx 0$. This situation occurs at approximately $h_{lo}^{max}(R_c) = R_c + 2R_b$, i.e. before the outer torus radius becomes R_c.

The local stretching energy $G_{str}^{loc}(h_{lo}) = \sigma \cdot \Delta A(h_{lo})$ considers the membrane area change $\Delta A(h_{lo})$, consisting of a spherical cap indentation (area $A_{cap}(h_{lo})$ at bead radius R_b) and a torus-ring like deformation with area $A_{tor}(h_{lo})$ with outer radius (R_c+R_b) and inner radius R_b relative to an approximately flat area $A_{flat}(h_{lo})$ (with radius R_c+R_b):

$$G_{str}^{loc}(h_{lo}) = \sigma \cdot \Delta A(h_{lo}) = \sigma \cdot \left(A_{cap}(h_{lo}) + A_{tor}(h_{lo}) - A_{flat}(h_{lo}) \right). \tag{13}$$

As shown in Supplementary Figs. S11 and S12, the area of the flat circle and the torus begin to decrease for $\theta > 90°$ such that $\Delta A(h_{lo}) \rightarrow 0$ at the length $h_{lo}^{\max}$, which is determined numerically, but can be approximated by $h_{lo}^{\max}(R_c) = R_c + 2R_b$.

The increase in cap plus torus area relative to the flat area with θ can be written as:

$$\begin{aligned} & A_{cap}(\theta, R_c) + A_{tor}(\theta, R_c) - A_{flat}(\theta, R_c) = \\ & = 2\pi \int_0^{\theta} \left(R_b \cdot \sin(\Theta) \right) + \left(R_c \left[\left(R_c + R_b \right) \cdot \sin(m \cdot \theta) - R_c \cdot \sin(\Theta) \right] \right) d\Theta - \left(\pi \left(R_c + R_b \right)^2 \cdot \sin(m \cdot \theta) \right) \end{aligned} \tag{14}$$

and is illustrated in Supplementary Fig. S11 at the bottom.

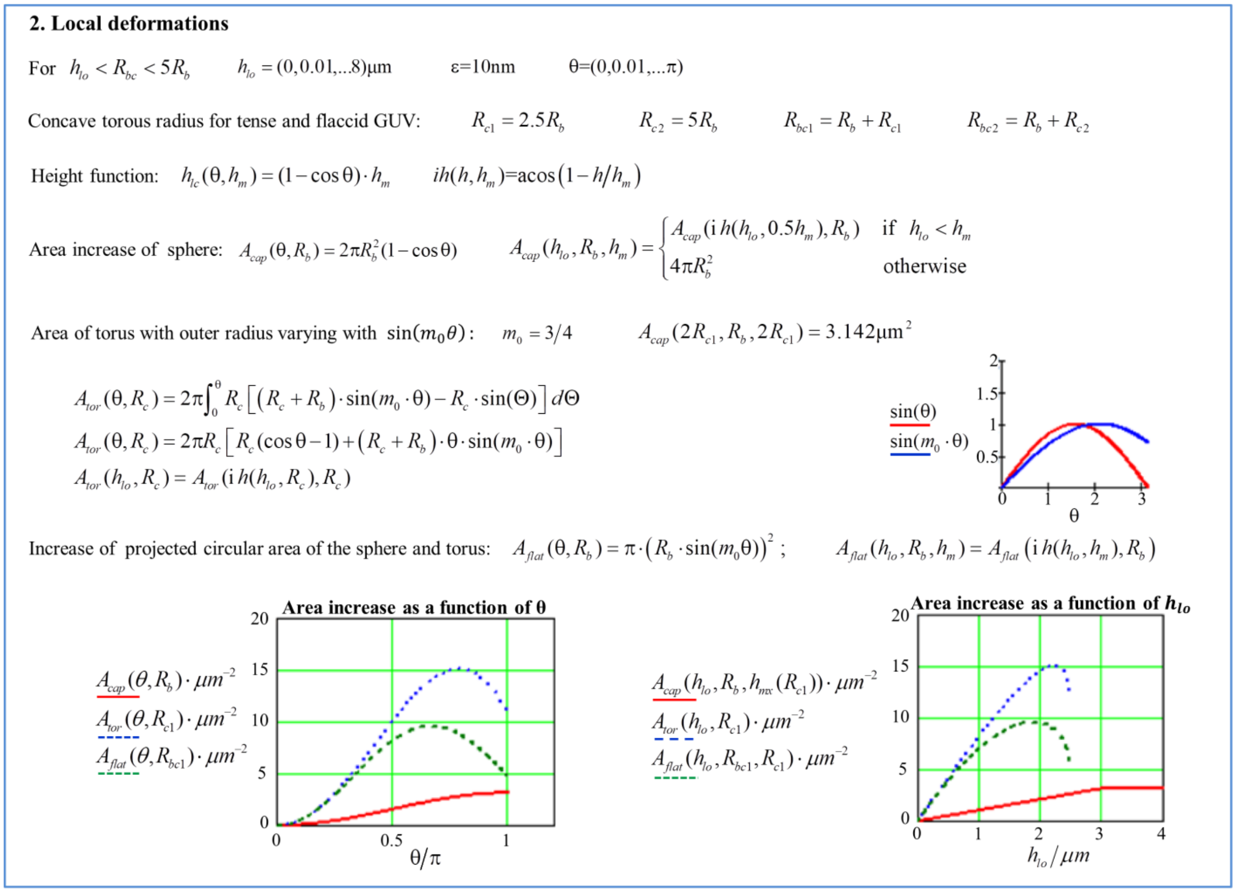

Supplementary Fig. S11: Area increase of spherical cap, torus and flat circular area. According to the right sketch in Supplementary Fig. S10 the radii of the three shapes increases and decreases according to (R_c+R_b)·sin(m_0·θ), where m_0 = 3/4.

The increase in membrane area with indentation height h_{lo} for the sphere, the torus and the projected flat area is shown in Supplementary Fig. S12. The increase $\Delta A(h_{lo})$ becomes zero at $h_{lo}^{\max}$ when $A_{cap}(h_{lo}^{\max}) + A_{tor}(h_{lo}^{\max}) = A_{flat}(h_{lo}^{\max})$.

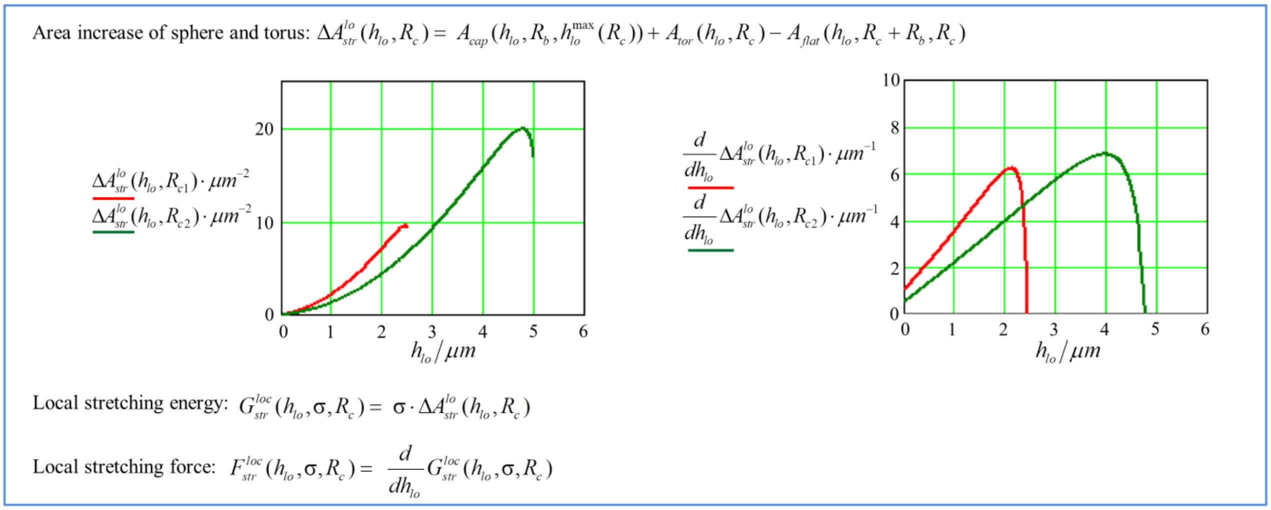

Supplementary Fig. S12: Area increase. Increase in membrane area ΔA with increasing indentation height h_{lo} for the sum of sphere, torus and projected flat areas. The slope $\frac{\partial}{\partial h_{lo}} \Delta A$ is proportional to the local stretching force (right).

The local bending energy $G_{ben}^{loc}(h_{lo})$ with bending rigidity K increases with the local surface areas of spherical cap and torus and their squared sum of curvatures in orthogonal directions:

$$G_{ben}^{loc}(h_{lo}) = \tfrac{1}{2} K \cdot \left(A_{cap}(h_{lo}) \cdot \tfrac{4}{R_b^2} + A_{tor}(h_{lo}) \cdot \left(\kappa_3(h_{lo}) + \kappa_4(h_{lo}) \right)^2 - A_{flat}(h_{lo}) \cdot 0 \right), \tag{15}$$

where $A_{cap}(h_{lo}) \cdot \frac{4}{R_b^2} = 8\pi \cdot p(h_{lo}) = 8\pi \frac{h_{lo}}{R_b + R_c}$. The flat area has a zero-curvature. The torus curvatures κ_3 and κ_4 are defined below in $G_{ben}^{loc}(\theta)$.

In particular, the θ dependent change in local bending energy by the torus formation can be described by

$$G_{ben}^{loc}(\theta) = \tfrac{1}{2} K \int_0^{\theta} \left(R_c \left[(R_c + R_b) \cdot \sin(\theta \cdot m) - R_c \cdot \sin(\Theta) \right] \right) \left(\tfrac{1}{R_c} + \left| \tfrac{-\sin(\Theta)}{R_c + R_b - R_c \cdot \sin(\Theta)} \right| \right)^2 d\Theta. \tag{16}$$

The results from the model for the increase in both local deformation energies (Supplementary Eqs. (13) and (15)) are displayed in Supplementary Fig. S13.

Local bending energy for spherical cap independent of radius: $G_{ben}^{cap}(\theta, K, R_c) = \pi K \int_0^{\theta} \left(\frac{2}{R_b}\right)^2 \cdot \left(\sin\Theta \cdot R_b^2\right) d\Theta = 4\pi K(1-\cos\theta)$

Bead fully wrapped after $h_{lo} = h_{max}(R_c)$: $G_{ben}^{cap}(h_{lo}, K, R_c) = \begin{cases} 8\pi K \dfrac{h_{lo}}{h_{\max}(R_c)} & \text{if } h_{lo} < h_{\max}(R_c) \\ 8\pi K & \text{otherwise} \end{cases}$

Local bending energy for torus: $G_{ben}^{tor}(\theta, K, R_c) = \pi K \int_0^{\theta} \left(R_c\left[(R_c + R_b)\cdot \sin(m_0 \cdot \theta) - R_c \cdot \sin(\Theta)\right]\right)\left(\frac{1}{R_c} + \left|\frac{-\sin(\Theta)}{R_c + R_b - R_c \cdot \sin(\Theta)}\right|\right)^2 d\Theta$

$$G_{ben}^{tor}(h_{lo}, K, R_c) = G_{ben}^{tor}(ih(h_{lo}, R_c), K, R_c)$$

$$G_{ben}^{loc}(h_{lo}, K, R_c) = G_{ben}^{cap}(h_{lo}, K, R_c) + G_{ben}^{tor}(h_{lo}, K, R_c)$$

Local numerical bending force: $F_{ben}^{loc}(h_{lo}, K, R_c) = \frac{d}{dh_{lo}} G_{ben}^{loc}(h_{lo}, K, R_c)$

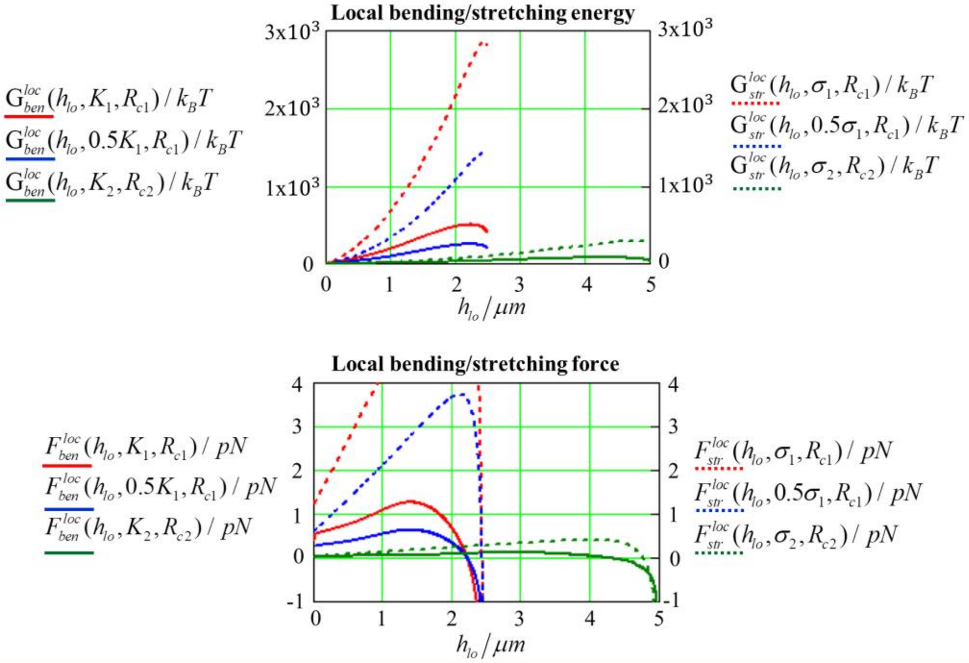

Supplementary Fig. S13: Local energies and forces for different indentation heights h_{lo}. Local stretching and bending energies (top, in k_BT) and forces (bottom, in pN) for three GUVs, with different radii, bending rigidities and surface tensions are compared for h_{lo} < 5 µm.

The results from the analytical model for the increase in local and global deformation energies are displayed in Supplementary Fig. S14 for three different GUVs with membrane parameters (σ_1, K_1, R_{g1}), (0.5σ_1, K_1, R_{g1}), and (σ_2, K_2, R_{g2}).

The indentation function $d(h_{lo}) = h_{lo} + h_{al} = h_{lo} + z(h_{lo})h_{lo}$ can be found by the zeros $z(h_{loc})$ of the corresponding force difference $F^{loc}(h_{lo}) - F^{glo}(h_{gl}) = 0$, such that $F^{loc}(h_{lo}) = F^{glo}(z(h_{lo}))$.

Although the deformation forces can be derived analytically, the minimum $z(h_{lo})$ of the force difference is found numerically (root command). $z(h_{lo})$ provides the ratio of the global indentation h_{gl} relative to the local indentation h_{lo}.

Total local energy: $G^{loc}(h_{lo},\sigma,K,R_c) = G^{loc}_{ben}(h_{lo},K,R_c) + G^{loc}_{str}(h_{lo},\sigma,R_c)$

Total local force: $F^{loc}(h_{lo},\sigma,K,R_c) = F^{loc}_{ben}(h_{lo},K,R_c) + F^{loc}_{str}(h_{lo},\sigma,R_c)$

$$F^{loc}(h_{lo},\sigma,K,R_c) = \frac{d}{dh_{lo}} G^{loc}(h_{lo},\sigma,K,R_c) \qquad F^{glo}(h_{gl},\sigma,K,R_g) = \frac{d}{dh_{gl}} G^{glo}(h_{gl},\sigma,K,R_g)$$

At the beginning G^{loc} rises faster than G^{glo} :

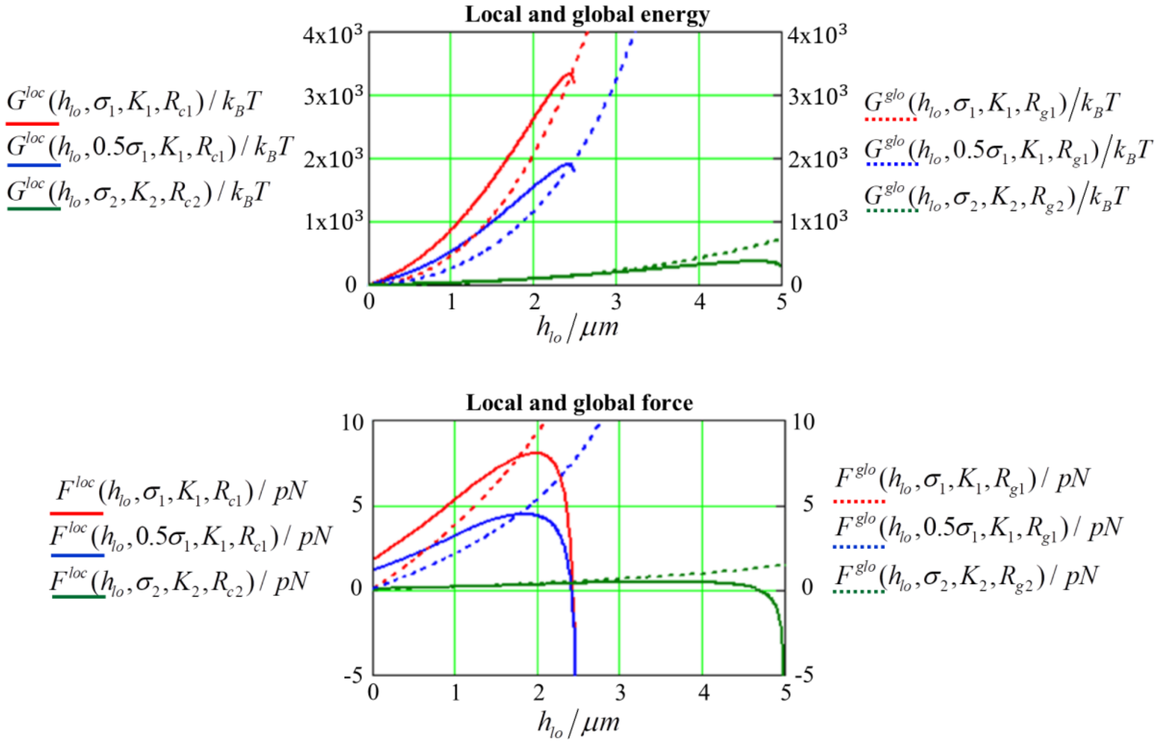

Supplementary Fig. S14: Comparison between local and global energies and forces as a function of the local indentation height h_{lo}.

It can be seen at the bottom right of Supplementary Fig. S15 that the total indentation $d(h_{lo})$ varies around $d(h_{lo}) \approx 2h_{lo}$ for a variety of membrane parameters.

In a serial connection of global and local spring forces, the force must be equal at each position such that $h_{tot}(F) = h_{gl}(F) + h_{lo}(F)$.

The derivation of energy dG/dh_{lo} corresponds to the force $F(h_{lo})$.

The position function which ensures that $F^{glo}(h_{gl}) = F^{loc}(h_{lo})$ is found:

$$F_{diff}(h_{gl}, h_{lo}, \sigma, K, R_c, R_g) = (F^{glo}(h_{gl}, \sigma, K, R_g) - F^{loc}(h_{lo}, \sigma, K, R_c)) \cdot 10^{12}$$

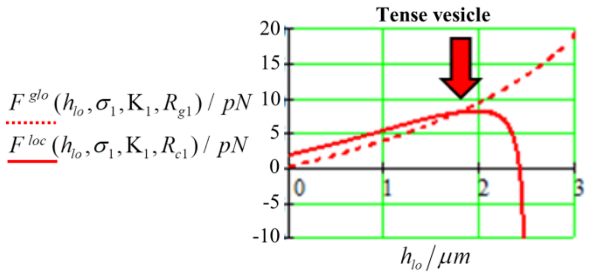

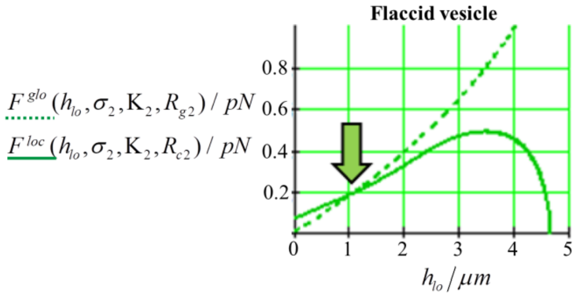

Find zeros of F_{diff} : $z(h_{lo}, \sigma, K, R_c, R_g) = root\left(F_{diff}(h_0, h_{lo}, \sigma, K, R_c, R_g), h_0\right)$ with $h_0 = 0.01 \mu m$

Total indentation : $d(h_{lo}) = h_{lo} + h_{gl} = h_{lo} + z(h_{lo}) \cdot h_{lo} = h_{lo} \cdot (1 + z(h_{lo}))$

$$d\left(h_{lo}, \sigma, K, R_c, R_g\right) = \begin{cases} h_{lo} + z(h_{lo}, \sigma, K, R_c, R_g) & \text{if } z(h_{lo}, \sigma, K, R_c, R_g) > h_0 \\ h_{lo} & \text{otherwise} \end{cases}$$

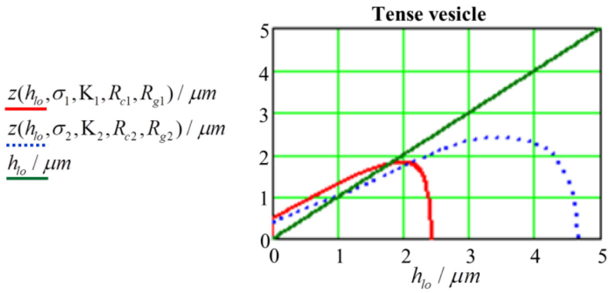

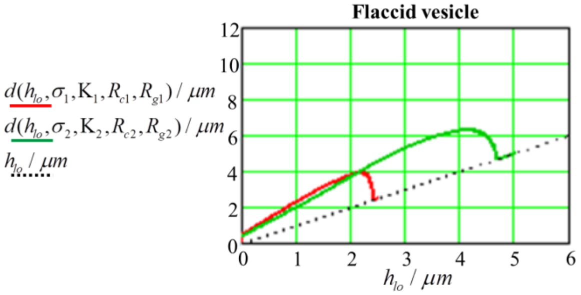

Supplementary Fig. S15: Finding equilibrium positions when global and local force are equal. Upper row: Change of the local and global forces with indentation h_{lo}. The red arrow marks the force equilibrium for a tense GUV, the green arrow for force equilibrium for a flaccid GUV. Lower row: Finding equilibrium positions by the zeros of the force differences. $z(h_{lo})$ is the ratio of the global h_{gl} relative to the local indentation h_{lo}. $d(h_{lo})$ describes the total bead indentation.

Supplementary Fig. S16 proves that the rescaling factor $z(h_{lo})$ is correct, leading to equal forces for all indentations h_{lo}.

Global and local forces are equal $F^{loc}(h_{lo}) = F^{glo}(z(h_{lo}))$ as required:

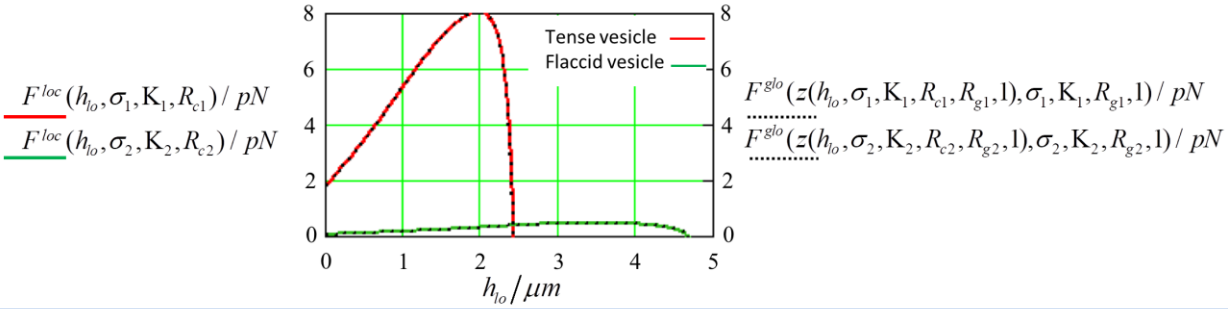

Supplementary Fig. S16: Local force profiles $F^{loc}(h_{lo})$ and global forces $F^{glo}(z(h_{lo}))$ are the same for all indentations h_{lo}.

The sum of the local energies and forces and the rescaled global energies and forces lead to the force profiles as a function of local indentation height h_{lo} are shown in Supplementary Fig. S17.

The total deformation energy force as a function of the indentation $d(h_{lo}) = h_{lo} + h_{al} = h_{lo} + z(h_{lo})h_{lo}$ leads to profiles of energy and force over longer extents, which are shown for the experimental values of a tense (top) and flaccid (bottom) vesicle in Supplementary Fig. S18. The profiles are set to zero by a step() functions for indentations where the force becomes negative, as visible in Supplementary Fig. S17 relative to Supplementary Fig. S18.

Total membrane deformation energy G_{mem} and total force F_{mem}:

$$G_{mem}(h_{lo},\sigma,K,R_c,R_g,w) = G^{loc}(h_{lo},\sigma,K,R_c) + G^{glo}(z(h_{lo},\sigma,K,R_c,R_g),\sigma,K,R_g) - G_{ad}(h_{lo},w)$$

$$F_{mem}(h_{lo},\sigma,K,R_c,R_g,w) = F^{loc}(h_{lo},\sigma,K,R_c) + F^{glo}(z(h_{lo},\sigma,K,R_c,R_g),\sigma,K,R_g) - F_{ad}(h_{lo},w)$$

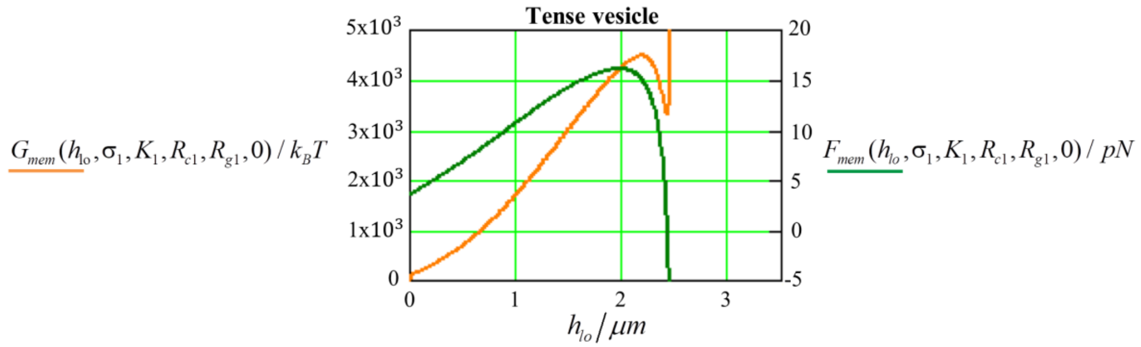

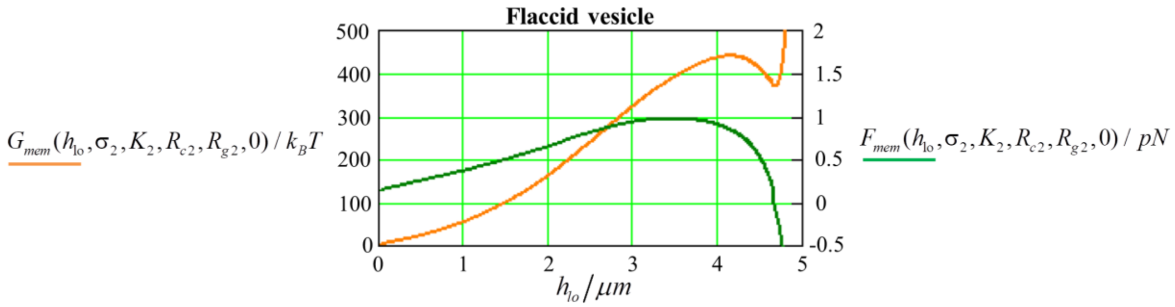

Supplementary Fig. S17: Total deformation energy and total force as a function of h_{lo}.

Total deformation energy and total force:

$$G_{mem}(h_{lo},\sigma,K,R_c,R_g,w) = G_{mem}(h_{lo},\sigma,K,R_c,R_g,w)\cdot \mathrm{step}(\mathrm{locup}(R_c)-h_{lo})$$

$$F_{mem}(h_{lo},\sigma,K,R_c,R_g,w) = (F_{mem}(h_{lo},\sigma,K,R_c,R_g,w) - F_{mem}(0.01\mu m,\sigma,K,R_c,R_g,w))\cdot \mathrm{step}(\mathrm{locup}(R_c)-h_{lo})$$

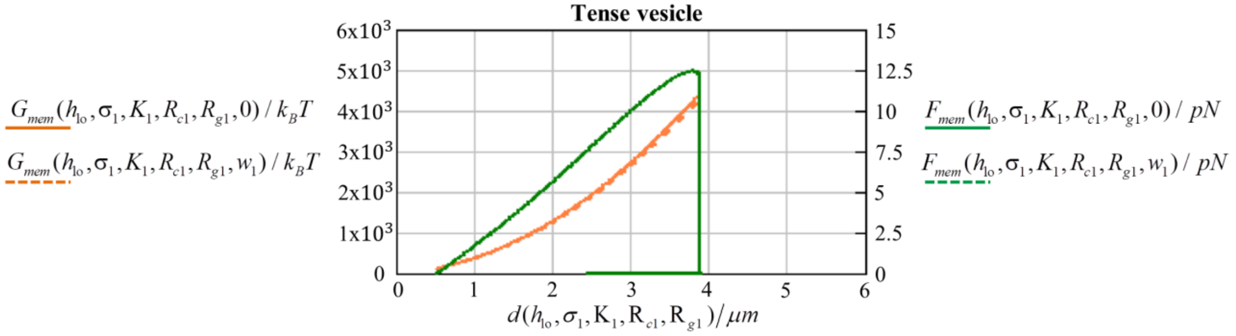

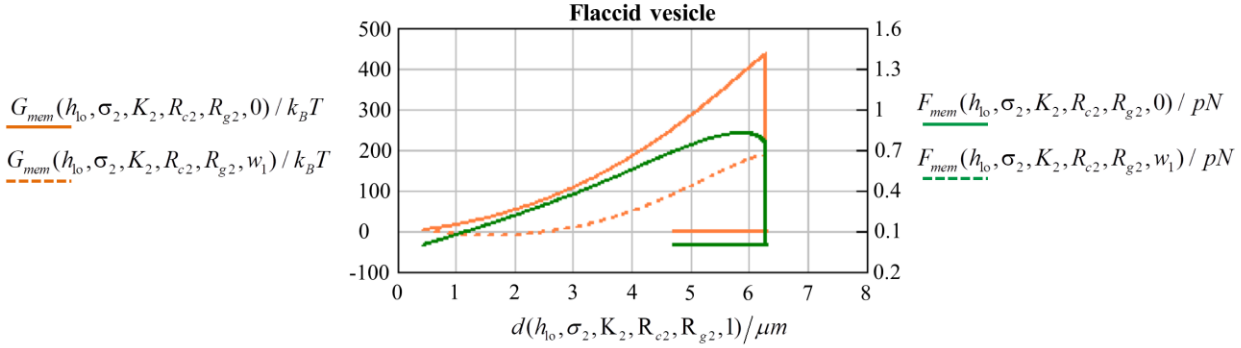

Supplementary Fig. S18: Total deformation energy and total force as a function of total indentation $d(h_{lo}) = h_{lo} + z(h_{lo})h_{lo}$.

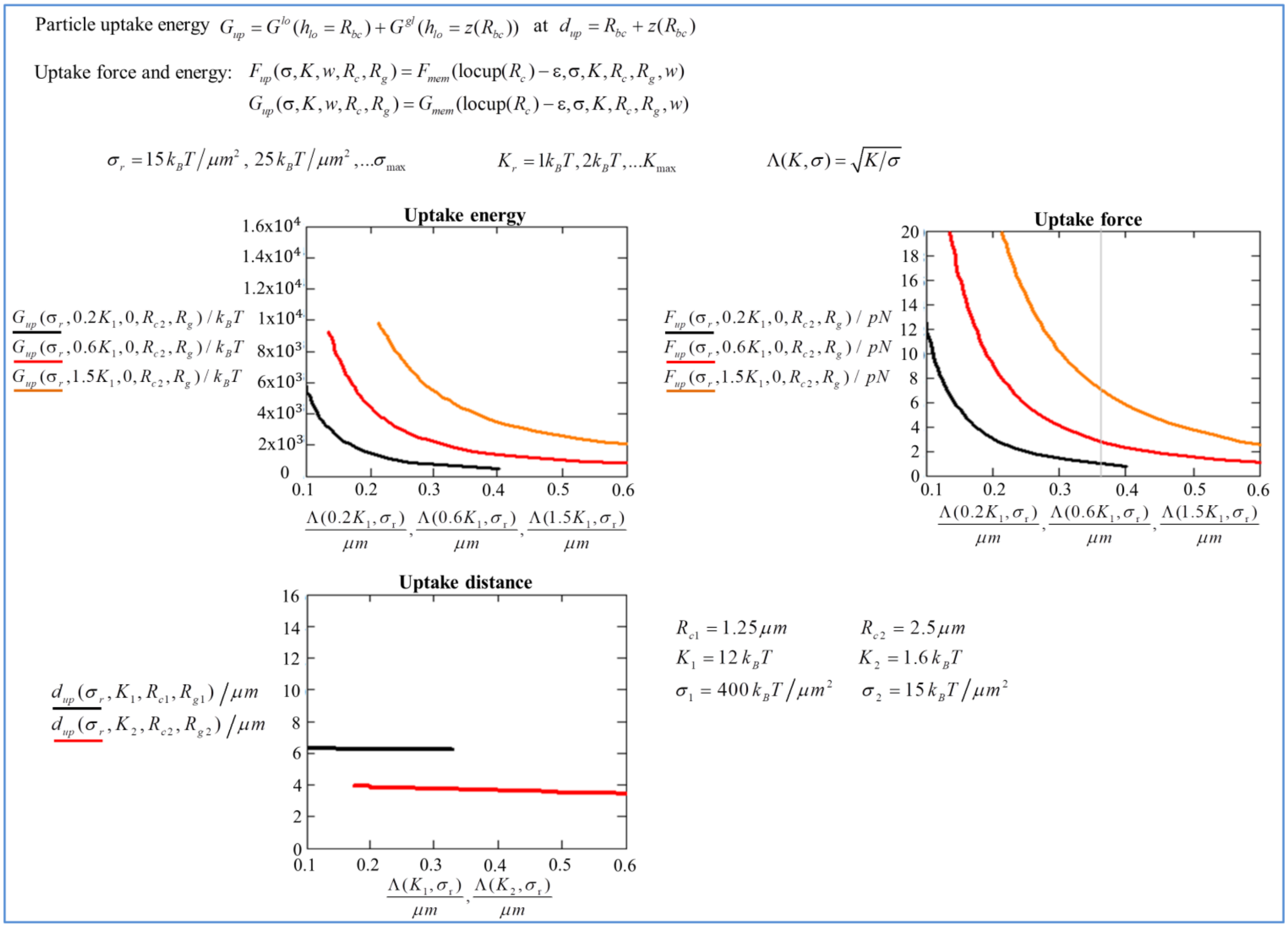

Supplementary Fig. S19: Calculated uptake energies and uptake forces for different membrane parameters Λ. $\Lambda(\sigma_r)$ is varied as a function of membrane tension σ_r, where three different bending rigidities K_r are used as parameter. The GUV radius is R_g = 10 µm and the torus radius is R_{c2} = 2.5 µm in the upper row, and R_{g1} = 7.5 µm with R_{c1} = 1.25 µm and R_{g2} = 13 µm with R_{c2} = 2.5 µm in for the calculated uptake lengths.

Supplementary References

1 Betz, T. & Sykes, C. Time resolved membrane fluctuation spectroscopy. *Soft Matter* **8**, 5317-5326, doi:10.1039/C2SM00001F (2012).

2 Helfrich, W. Elastic properties of lipid bilayers: theory and possible experiments. *Zeitschrift fur Naturforschung. Teil C: Biochemie, Biophysik, Biologie, Virologie* **28**, 693-703 (1973).

3 Seifert, U. Configurations of fluid membranes and vesicles. *Advances in physics* **46**, 13-137 (1997).

4 Meinel, A., Trankle, B., Romer, W. & Rohrbach, A. Induced phagocytic particle uptake into a giant unilamellar vesicle. *Soft Matter* **10**, 3667-3678, doi:10.1039/c3sm52964a (2014).
