# Supplementary material for: Thermal fluctuations of the lipid membrane determine particle uptake into Giant Unilamellar Vesicles
Source: Nat Commun. 2023 Jan 4;14:65. doi: 10.1038/s41467-022-35302-5 (PMC9813155; doi:10.1038/s41467-022-35302-5)
Supplement: Supplementary file 3 — Description of Additional Supplementary Files [file 41467_2022_35302_MOESM3_ESM.pdf]

Description of Additional Supplementary Files

File Name: Supplementary Movie 1
Description: Flaccid GUV only

File Name: Supplementary Movie 2
Description: Tense GUV only

File Name: Supplementary Movie 3
Description: Flaccid GUV with bead plus fluctuation analysis

File Name: Supplementary Movie 4
Description: Tense GUV with bead plus fluctuation analysis

File Name: Supplementary Movie 5
Description: Flaccid GUV fluorescence 3D animated

File Name: Supplementary Movie 6
Description: Tense GUV fluorescence 3D animated

File Name: Supplementary Movie 7
Description: Bead-deforms-membranetorus-like
