# Supplementary material for: Thermal fluctuations of the lipid membrane determine particle uptake into Giant Unilamellar Vesicles
Source: Nat Commun. 2023 Jan 4;14:65. doi: 10.1038/s41467-022-35302-5 (PMC9813155; doi:10.1038/s41467-022-35302-5)
Supplement: Supplementary file 11 — Reporting Summary [file 41467_2022_35302_MOESM11_ESM.pdf]

nature portfolio

Corresponding author(s): Alexander Rohrbach

Last updated by author(s): Nov 17, 2022

Reporting Summary

Nature Portfolio wishes to improve the reproducibility of the work that we publish. This form provides structure for consistency and transparency in reporting. For further information on Nature Portfolio policies, see our Editorial Policies and the Editorial Policy Checklist.

Statistics

For all statistical analyses, confirm that the following items are present in the figure legend, table legend, main text, or Methods section.

n/a	Confirmed	
☐	☒	The exact sample size (*n*) for each experimental group/condition, given as a discrete number and unit of measurement
☐	☒	A statement on whether measurements were taken from distinct samples or whether the same sample was measured repeatedly
☒	☐	The statistical test(s) used AND whether they are one- or two-sided<br>*Only common tests should be described solely by name; describe more complex techniques in the Methods section.*
☒	☐	A description of all covariates tested
☒	☐	A description of any assumptions or corrections, such as tests of normality and adjustment for multiple comparisons
☐	☒	A full description of the statistical parameters including central tendency (e.g. means) or other basic estimates (e.g. regression coefficient) AND variation (e.g. standard deviation) or associated estimates of uncertainty (e.g. confidence intervals)
☒	☐	For null hypothesis testing, the test statistic (e.g. *F*, *t*, *r*) with confidence intervals, effect sizes, degrees of freedom and *P* value noted<br>*Give P values as exact values whenever suitable.*
☒	☐	For Bayesian analysis, information on the choice of priors and Markov chain Monte Carlo settings
☒	☐	For hierarchical and complex designs, identification of the appropriate level for tests and full reporting of outcomes
☒	☐	Estimates of effect sizes (e.g. Cohen's *d*, Pearson's *r*), indicating how they were calculated

Our web collection on statistics for biologists contains articles on many of the points above.

Software and code

Policy information about availability of computer code

Data collection	Python Code for experimental setup control and measurement data collection and MathCad Version15 for simulations
Data analysis	Python, Igor Pro Version 9 and ImageJ 64-bit Java 8 for processing and analysis of measurement data

For manuscripts utilizing custom algorithms or software that are central to the research but not yet described in published literature, software must be made available to editors and reviewers. We strongly encourage code deposition in a community repository (e.g. GitHub). See the Nature Portfolio guidelines for submitting code & software for further information.

Data

Policy information about availability of data

All manuscripts must include a data availability statement. This statement should provide the following information, where applicable:

- Accession codes, unique identifiers, or web links for publicly available datasets
- A description of any restrictions on data availability
- For clinical datasets or third party data, please ensure that the statement adheres to our policy

The data generated in this study are provided with this paper in the Source Data file. Analysis codes are available from the corresponding author upon reasonable request.

Human research participants

Policy information about studies involving human research participants and Sex and Gender in Research.

Reporting on sex and gender	N/A
Population characteristics	N/A
Recruitment	N/A
Ethics oversight	N/A

Note that full information on the approval of the study protocol must also be provided in the manuscript.

Field-specific reporting

Please select the one below that is the best fit for your research. If you are not sure, read the appropriate sections before making your selection.

[x] Life sciences [ ] Behavioural & social sciences [ ] Ecological, evolutionary & environmental sciences

For a reference copy of the document with all sections, see nature.com/documents/nr-reporting-summary-flat.pdf

Life sciences study design

All studies must disclose on these points even when the disclosure is negative.

Sample size	Sample sizes were determined according to the number of complete uptake experiments achieved.<br>The successful uptake were determined by the morphological and mechanical characteristics of individual vesicles (tension and membrane bending rigidity) along with the maximum optical force driving the particle internalization. We used low optical forces in order to preserve the integrity of lipids. We performed experiments resulting in N=32 independent DOPC vesicles(2-Dioleoyl-sn-glycero-3-phosphocholine), N=13 Gb3-LecA vesicles (glycosphingolipid Gb3 - Lectin A), and N=5 Bio-Strep vesicles (Biotin - Streptavidin). These numbers of experiments clearly pointed out a correlation between the force/ energy required to internalize the particle and the different mechanical properties and fluctuation parameters of the tested vesicles.
Data exclusions	No data was excluded
Replication	Our samples were produced following the same protocol. To avoid the possibility of undesired lipids attaching to the particle due to repeated experiments on the same vesicle, the data presented were obtained from N=32 independent DOPC vesicles(2-Dioleoyl-sn-glycero-3-phosphocholine), N=13 Gb3-LecA vesicles (glycosphingolipid Gb3 - Lectin A), and N=5 Bio-Strep vesicles (Biotin - Streptavidin) indented only once.
Randomization	n/a - since there was no group allocation
Blinding	n/a - since there was no group allocation

Reporting for specific materials, systems and methods

We require information from authors about some types of materials, experimental systems and methods used in many studies. Here, indicate whether each material, system or method listed is relevant to your study. If you are not sure if a list item applies to your research, read the appropriate section before selecting a response.

Materials & experimental systems

n/a	Involved in the study	
[ ]	[x]	Antibodies
[x]	[ ]	Eukaryotic cell lines
[x]	[ ]	Palaeontology and archaeology
[x]	[ ]	Animals and other organisms
[x]	[ ]	Clinical data
[x]	[ ]	Dual use research of concern

Methods

n/a	Involved in the study	
[x]	[ ]	ChIP-seq
[x]	[ ]	Flow cytometry
[x]	[ ]	MRI-based neuroimaging

Antibodies

Antibodies used	FSL-Biotin, Sigma Aldrich F9182.<br>Streptavidin conjugated microspheres, Polyscience Inc.<br>Purified Gb3 extracted from red blood cells were supplied from Matreya.<br>Recombinat LecA produced from Escherichia coli labelled with Biotin NHS ester.<br>L-ALPHA-PHOSPHATIDYLCHOLINE, DIOLEOYL, Product Number: P6354-25MG Sigma.<br>Texas Red 1,2-dihexadecanoyl-sn-glycero-3-phosphaethanolamine (Tx-Red DHPE) from Life Technologies
Validation	The binding of LecA after biotinylation to Gb3 receptors was examined by means of Gb3-functionalized vesicles by fluorescently labeled streptavidin molecules (Figs S4 and S5 DOI: 10.1039/d0nr07726g).<br>Gb3-LecA adhesion specificity was tested by detachment experiments (Fig 4 DOI: 10.1039/d0nr07726g).
